# Explainable Multimodal Graph Isomorphism Network for Interpreting Sex Differences in Adolescent Neurodevelopment

**DOI:** 10.3390/app14104144

**Published:** 2024-05-14

**Authors:** Binish Patel, Anton Orlichenko, Adnan Patel, Gang Qu, Tony W. Wilson, Julia M. Stephen, Vince D. Calhoun, Yu-Ping Wang

**Affiliations:** 1 Biomedical Engineering Department, Tulane University, New Orleans, LA 70118, USA; 2 Department of Business Administration, University of Illinois Urbana-Champaign, Champaign, IL 61820, USA; 3 Institute for Neuroscience, Boys Town National Research Hospital, Boys Town, NE 68010, USA; 4 Mind Research Network, Albuquerque, NM 87106, USA; 5 Tri-Institutional Center for Translational Research in Neuroimaging and Data Science (TReNDS) Georgia State University, Georgia Institute of Technology, Emory University, Atlanta, GA 30030, USA

**Keywords:** deep learning, graph neural network, interpretability, multi-modality, multi-paradigm, sex differences

## Abstract

**Background:**

A fundamental grasp of the variability observed in healthy individuals holds paramount importance in the investigation of neuropsychiatric conditions characterized by sex-related phenotypic distinctions. Functional magnetic resonance imaging (fMRI) serves as a meaningful tool for discerning these differences. Among deep learning models, graph neural networks (GNNs) are particularly well-suited for analyzing brain networks derived from fMRI blood oxygen level-dependent (BOLD) signals, enabling the effective exploration of sex differences during adolescence.

**Method:**

In the present study, we introduce a multi-modal graph isomorphism network (MGIN) designed to elucidate sex-based disparities using fMRI task-related data. Our approach amalgamates brain networks obtained from multiple scans of the same individual, thereby enhancing predictive capabilities and feature identification. The MGIN model adeptly pinpoints crucial subnetworks both within and between multi-task fMRI datasets. Moreover, it offers interpretability through the utilization of GNNExplainer, which identifies pivotal sub-network graph structures contributing significantly to sex group classification.

**Results:**

Our findings indicate that the MGIN model outperforms competing models in terms of classification accuracy, underscoring the benefits of combining two fMRI paradigms. Additionally, our model discerns the most significant sex-related functional networks, encompassing the default mode network (DMN), visual (VIS) network, cognitive (CNG) network, frontal (FRNT) network, salience (SAL) network, subcortical (SUB) network, and sensorimotor (SM) network associated with hand and mouth movements. Remarkably, the MGIN model achieves superior sex classification accuracy when juxtaposed with other state-of-the-art algorithms, yielding a noteworthy 81.67% improvement in classification accuracy.

**Conclusion:**

Our model’s superiority emanates from its capacity to consolidate data from multiple scans of subjects within a proven interpretable framework. Beyond its classification prowess, our model guides our comprehension of neurodevelopment during adolescence by identifying critical subnetworks of functional connectivity.

## 1. Introduction

The critical time period for the onset of mental illness is adolescence. Neuroimaging can help to inform how aberrations in brain circuits might be present in psychiatric disorders and their development during adolescence [1–5]. Utilizing functional neuroimaging studies to understand sex differences in healthy brains can provide an important foundation to guide sex-specific treatments [6–9]. Functional magnetic resonance imaging (fMRI) is a neuroimaging method that measures the hemodynamic changes in regional brain oxygenation. By enhancing the field’s understanding of sex differences in healthy individuals, we can develop a more thorough understanding of neurodevelopmental disorders that exhibit phenotypic differences between sexes [10,11]. Machine learning-based methods, such as graph neural networks (GNNs), can be employed in the context of fMRI data to proficiently unveil new biomarkers that underlie developmental differences in sex, age, and cognition [12–21].

The graph isomorphism network (GIN) is a specialized type of graph neural network that has been shown to achieve superior discriminative power among GNNs for classification tasks. Ultimately, the node embeddings acquired through the GIN classifier serve the purpose of generating the embedding for an entire graph. Additionally, GIN is regarded as a dual representation of the convolutional neural network [22]. In the realm of graph theory, the graph isomorphism problem is centered on the determination of whether two graphs exhibit topological equivalence. Two graphs are considered isomorphic if a node mapping exists that preserves node adjacencies, signifying that an edge connecting a pair of nodes in the first graph is mirrored in the second graph in the same manner. Essentially, isomorphic graphs share identical connectivity and vary solely due to node permutation. The network enacts the aggregate and combine functions by summing the node features. For the graph-level readout, the embedded node features from each layer are summated and subsequently concatenated to yield the ultimate graph feature. The authors contend that the proposed network architecture has the capacity to learn an injective mapping of the function g, potentially endowing the model with similar capabilities to the Weisfeiler–Lehman (WL) test for graph classification tasks [22]. In contrast to existing GNNs, which utilize non-injective neighbor aggregation and thus possess limited discriminative power, GIN employs injective neighbor aggregation and is as discriminative as the WL graph isomorphism test [22].

In recent times, there has been an increasing interest in the utilization of graph-based deep learning models in diverse fMRI investigations, encompassing areas such as disease prediction [23] and biomarker identification [24]. The use of graph deep learning models proves advantageous in brain network analysis due to their ability to directly process graph-structured data as the input. This characteristic makes them well-suited for exploring and understanding the intricate connectivity patterns within the brain. Li et al. proposed the BrainGNN framework and utilized fMRI data and graph attention mechanisms to discover novel biomarkers for autism spectrum disorder [24]. Some of the limitations of the BrainGNN method include the extent of interpretability, computational complexity, and generalizability.

Furthermore, Tang et al. developed a novel approach for brain network analysis using contrastive learning and hierarchical signed graph pooling [25]. Their proposed method aims to capture meaningful representations of brain networks by leveraging the inherent structure and connectivity patterns in the data. Some of the limitations of the study include challenges with interpretability, computational complexity, and a lack of comparison with existing state-of-the-art deep learning techniques. In the contrastive learning approach, the authors did not discuss the interpretability of the obtained features or their clinical relevance. Understanding the underlying mechanisms driving the learned representations could provide valuable insights into brain function and pathology. Additionally, Cui et al. presented BrainGB, a benchmark dataset and evaluation framework for brain network analysis using GNNs [26]. The authors addressed the need for standard evaluation in the field by providing a comprehensive benchmark that covers multiple tasks and datasets. A major limitation of the BrainGB framework is its simplified representation of brain networks, which primarily focuses on using brain regions as nodes in the constructed brain networks. However, brain connectivity is a complex and multi-modal phenomenon involving various levels of granularity and different types of connections. Additionally, the authors of BrainGB do not discuss the interpretability of the GNN models and the insights gained from their predictions [26]. Understanding the underlying factors contributing to the model’s decisions as well as the meaningfulness of the learned representations would enhance the practical utility of the benchmark.

Motivated by the above observations, we propose an interpretable end-to-end multi-modal graph isomorphism network (MGIN) framework to integrate multiple paradigms of fMRI, which incorporates information about signals in each brain region of interest and functional connectivity. Existing GIN methods applied to fMRI connectivity networks do not utilize data to understand the relation information both within and between modalities. Previous methods utilizing GIN only use a single modality (e.g., resting state fMRI data) for the classification tasks without discussing specific regions of interest and subnetwork structures relevant to understanding the findings. To validate the model, we applied MGIN to a large publicly accessible dataset, specifically, the Philadelphia Neurodevelopmental Cohort (PNC). Our results show that the algorithm is robust and can extract important subnetwork connections in multi-modal data. In our data analysis, we integrate multitask data using our proposed algorithm and demonstrate better accuracy in classifying sex differences than using a single task. Compared to other state-of-the-art models, our algorithm achieves better accuracy and can detect important intra- and inter-network connections during the different stages of adolescent neurodevelopment [27–29].

The main contributions of our proposed method include the following:

1. Our method alleviates the problem of predetermining the best features from connectivity networks, which are often ambiguous due to the high-dimensional nature of neuroimaging data. To overcome these challenges, our framework utilizes all connectivity networks without pre-determining features in order to mitigate the impact of high dimensionality.
2. We propose a generalizable model to accurately predict sex between the five different stages of adolescence. Our method is robust, multi-task, multi-modal, and generalizable to other applications and modalities.
3. Based on graph isomorphism, our graph neural network classifier can be employed for multigraphs characterized by varying nodes and edges, all the while acquiring local graph knowledge without being restricted to the entirety of the graph. By emphasizing the learning of local graph information, the model can effectively leverage the inherent structure and relationships within the graph to perform classification tasks.
4. Lastly, our MGIN model is interpretable by applying the GNNExplainer [30] method to understand important subnetwork connections in the brain during the five stages of adolescence, thereby providing an insight on brain network mechanisms underlying development. Our framework illustrates important regions of interest as well as subnetwork connections during neurodevelopment.

The subsequent sections of this manuscript are organized as follows. Section 2 reviews the graph isomorphism network and introduces the proposed multi-modal graph isomorphism network method. In Section 3, we provide the experimental outcomes and engage in discussions pertaining to the analysis of PNC data, aimed at elucidating the various phases of adolescence. Finally, Section 4 serves as the concluding segment of this manuscript. It is worth noting that a preliminary iteration of this research has been documented in a prior conference proceeding [31].

## 2. Methodology

### 2.1. Data Collection and Pre-Processing

The dataset analyzed in this study is provided by the Philadelphia Neurodevelopmental Cohort (PNC). PNC is a collaboration between the Children’s Hospital of Philadelphia and the University of Pennsylvania, funded by the National Institute of Mental Health (NIMH) to characterize brain and behavioral interactions. The PNC consists of over 800 healthy subjects aged from 8 to 22 years. The fMRI tasks adopted in this study are emotion identification (emoid) and working memory (nback) tasks. All MRI scans were conducted using a single 3T Siemens TIM Trio whole-body scanner. During the task, participants were instructed to categorize emotions depicted in facial expressions, encompassing happiness, anger, sadness, fear, and neutrality. The total duration of the scanning session was 10.5 min. The blood oxygenation level-dependent (BOLD) signal was captured utilizing a whole-brain, single-shot, multislice, gradient-echo echoplanar sequence comprising 124 volumes (372 s) [32]. Standard pre-processing procedures were implemented utilizing SPM12 (http://www.fil.ion.ucl.ac.UK/spm/software/spm12/ (accessed on 1 March 2022)), including motion correction, spatial normalization to standard MNI space, and spatial smoothing employing a 3 mm full width at half maximum Gaussian kernel [33]. We adhered to a standard pre-processing pipeline as outlined in our previous works [13,14] using SPM12.

**Figure 1.**
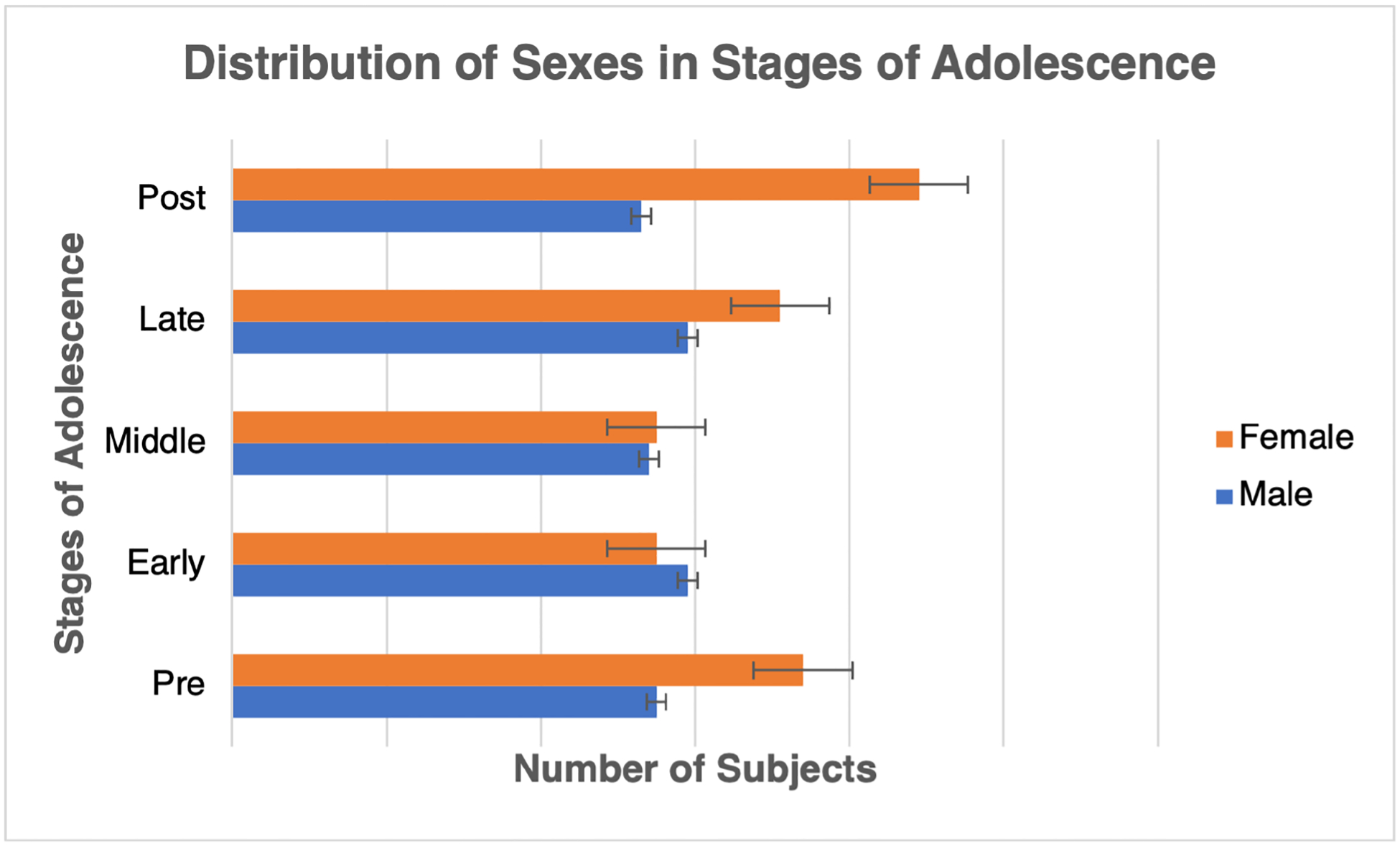
The distribution of sexes in the various stages of adolescence.

The Power template [34] is used to divide the brain into 264 regions of interest (ROIs) to parcellate the BOLD signal and build the connectivity matrix using the Pearson correlation. Within the scope of this research, a sample of 622 subjects was selected from the overall pool of 800 participants. This specific subset was chosen due to the availability of both emotion identification (emoid) and working memory (nback) paradigms within the fMRI scan. The male and female subjects were divided into five groups, each representing a stage related to adolescent development as shown in Figure 1. The frequently used notation is listed in Table 1, and the group division information is listed in Table 2 [35].

The MGIN model was compared to other state-of-the-art algorithms, i.e., SVM, GIN [22], BrainGNN [24], M-GCN [36], MVGCN [37], MLP, and MGIN. SVM is a baseline supervised machine learning algorithm that is commonly used for classification analysis, which constructs a hyperplane in a high-dimensional space to separate data points into different classes. GIN [22] is a deep learning architecture that consists of multiple graph convolution layers followed by fully connected layers by applying a transformation to each node’s features based on its neighborhood. BrainGNN [24] is a graph neural network architecture designed for brain network analysis that incorporates brain-specific prior knowledge into the neural network structure to improve the accuracy of the classification. M-GCN [36] is a multi-modal deep learning architecture that integrates functional and structural connectomics data to predict multidimensional phenotypic characterization to capture the complex relationships between brain regions at different levels of organization. MVGCN [37] is a graph convolutional network designed for multi-view data integration that can learn a shared representation across multiple views of the data and perform classification on the integrated representation. MLP is a neural network architecture that consists of multiple layers of neurons, where each neuron applies a non-linear function to the weighted sum of its inputs. MGIN is our proposed multi-modal deep learning framework used for understanding neurodevelopment.

### 2.2. Overview of the Pipeline

**Figure 2.**
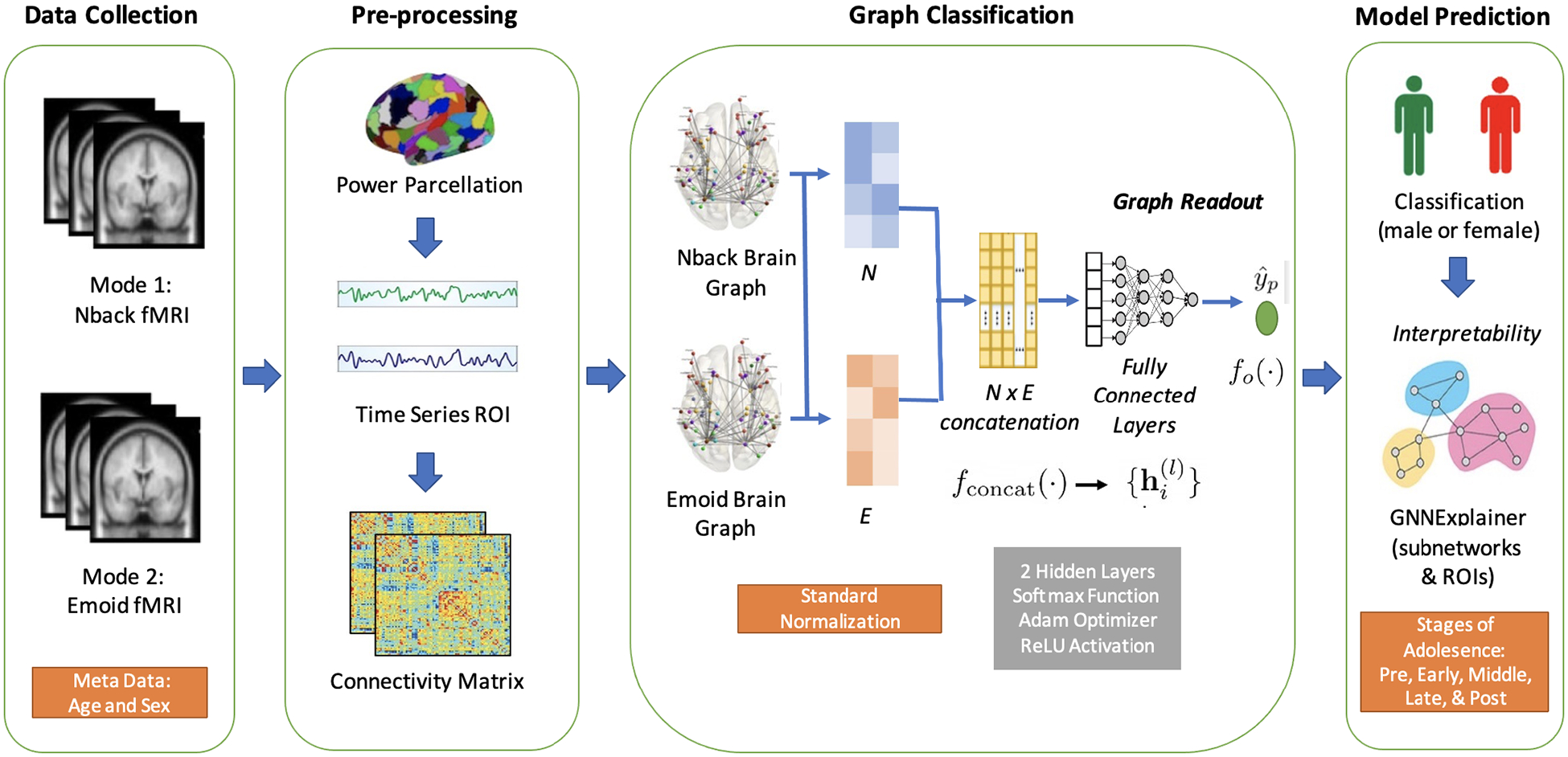
Overview of the proposed framework. The fMRI time-series data were preprocessed and divided into 264 regions of interest (ROIs) based on the Power coordinates template. Then, the mean BOLD time course data were extracted, and the Pearson correlation coefficient between ROIs was calculated and represented as a functional connectivity matrix. Then, the functional network graph was input to our MGIN model to classify sex. Lastly, the GNNExplainer method was applied to identify critical subnetwork structures and to identify important ROIs.

The summary of the analytic approach is provided in Figure 2. For each subject, the fMRI time series data were pre-processed, and the whole brain was divided into 264 regions of interest (ROIs) based on the Power coordinates template [34]. The mean BOLD time course data were extracted from each brain region, and the Pearson correlation coefficient between ROIs was calculated and represented as a functional connectivity matrix. Then, the functional network graph was input to our proposed MGIN model to classify sex groups based on age. Lastly, the GNNExplainer method was applied to identify critical subnetwork structures and to identify important connections between the functional networks [30].

### 2.3. Graph Isomorphism Network (GIN)

The graph isomorphism network (GIN) is formulated to accept networks as its input. Xu et al. introduced GIN as a specific instance of spatial GNN well-suited for tasks involving graph classification [22]. The network operationalizes the aggregate and combine functions by summing the node features, as depicted in Equation (1) below.

(1) $$h_{v}^{\left(k\right)}=MLP^{\left(k\right)}\left(\left(1+\epsilon^{\left(k\right)}\right)\cdot h_{v}^{\left(k-1\right)}+\sum_{u\in N(v)}h_{u}^{\left(k-1\right)}\right)$$

In Equation (1), $\epsilon^{\left(k\right)}$ represents a parameter that is adaptable at the kth layer, while MLP denotes a multi-layer perceptron incorporating non-linearities. The notation $h_{v}^{\left(k\right)}$ signifies the feature vector at the kth layer for the vth node. The feature vector corresponds to layer k, where it integrates the previous node feature $h_{v}^{\left(k-1\right)}$ with aggregated node features to output the node feature of the current kth layer, denoted as $h_{v}^{\left(k\right)}$. For a graph-level readout, the embedded node features from each layer are summed and subsequently concatenated to derive the final graph feature $h_{G}$, as illustrated in Equations (2) and (3) [38].

(2) $$h_{G}^{\left(k\right)}=\text{sum}\left(h_{0}^{\left(k\right)},h_{1}^{\left(k\right)},\ldots ,h_{N}^{\left(k\right)}\right)$$

(3) $$h_{G}=\text{concatenate}\left({\text{h}}_{\text{G}}^{\left(\text{k}\right)}|k=0,1,\ldots ,K\right)$$

Xu et al. asserted that the proposed network architecture has the capacity to acquire an injective mapping of the function g, potentially endowing the model with comparable effectiveness to the WL test for tasks involving graph classification [22,38].

(4) $$h_{v}^{\left(k\right)}=\emptyset \left(h_{v}^{\left(k-1\right)},f\left(\left\{h_{u}^{\left(k-1\right)}:u\;\epsilon \;N(v)\right\}\right)\right)$$

In Equation (4), $h_{v}^{\left(k\right)}$ is the feature vector of node v at the kth layer or iteration. N(v) is the set of nodes adjacent to v, and the equation aggregates and updates node features, where the function f operates on a multiset of node features and ϕ is the injective function. Furthermore, the WL test employs a pre-defined injective hash function g to update the node labels $l_{v}$:

(5) $$l_{v}^{\left(k\right)}=g\left(l_{v}^{\left(k-1\right)},\left\{l_{u}^{\left(k-1\right)}u\;\epsilon \;N(v)\right\}\right)$$

### 2.4. Multi-Modal Graph Isomorphism Network (MGIN)

**Figure 3.**
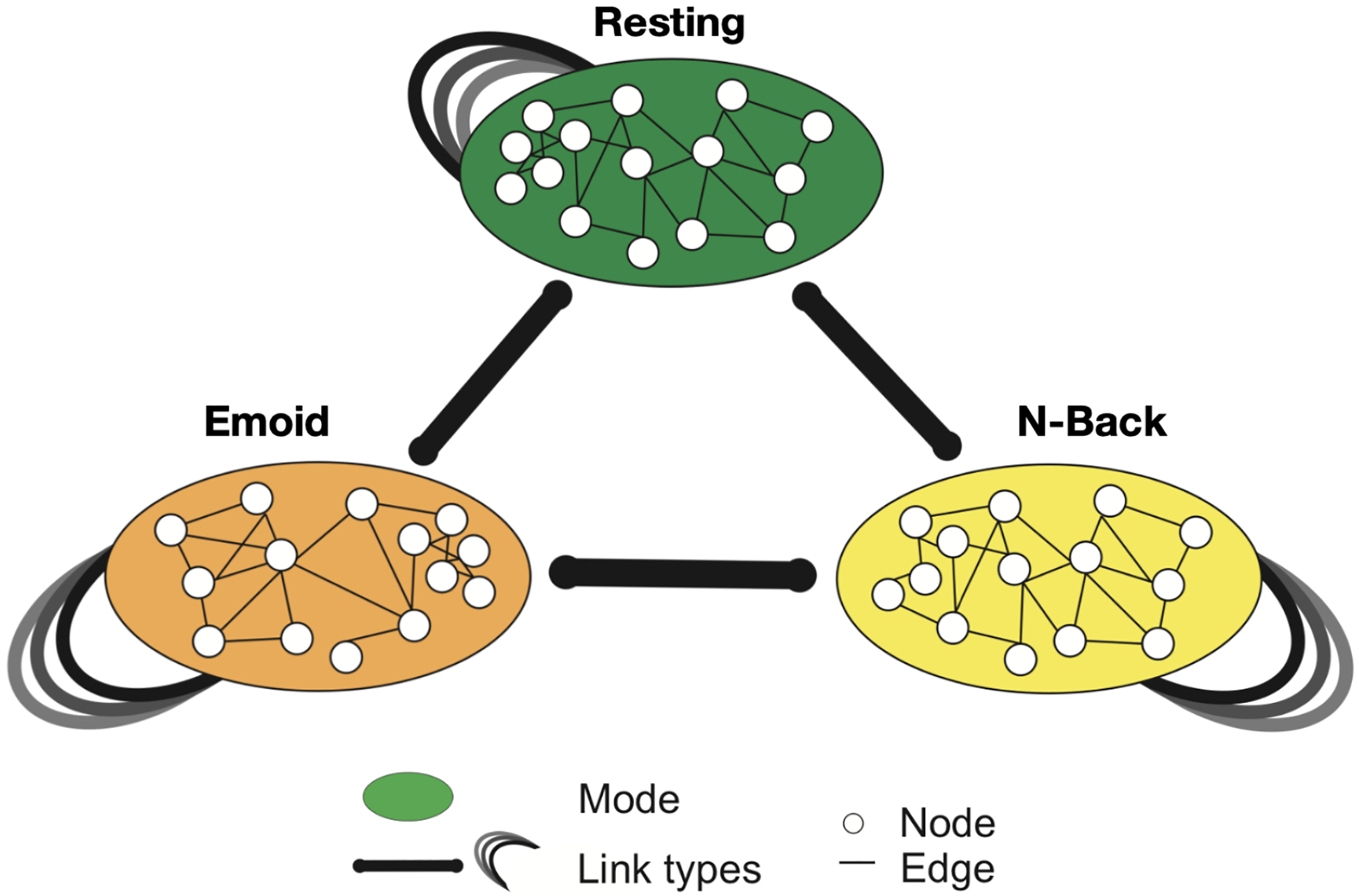
The proposed framework for integrating the multi-paradigm fMRI data to understand the interactome.

We next propose our multi-modal graph isomorphism network (MGIN) to combine the complementary information from multiple fMRI paradigms, which is then used to classify sex groups during adolescence. We proposed the MGIN framework for integrating the multi-paradigm fMRI data to understand the interactome, as shown in Figure 3. The MGIN model comprises multiple sets of nodes, referred to as modes, which individually represent different types of entities. These modes are interconnected through edges, facilitating connections between nodes within a mode as well as across different modes. The modes in our model are emotion identification and working memory fMRI task. The nodes are the functional connectivity networks for each subject [39]. In this study, each individual’s brain network is considered a single graph. The connections between regions of interest (ROIs) in the network are represented by the absolute value of the Pearson correlation coefficient between the time- eries of each pair of ROIs. The graph is defined as an undirected multigraph with G={V,E}. In this study, a two-layer GIN is utilized to acquire the graph embedding for an individual in each modality. For the vertices, we concatenated the features and used the Pearson correlation-based functional connectivity graph to construct the brain functional network. The Pearson correlation coefficient, which assesses the relationship between two variables denoted as X and Y, is computed utilizing the subsequent formula:

(6) $$P=\frac{cov(X,Y)}{\sigma_{X}\cdot \sigma_{Y}}$$

After that, we applied the Box-Cox transformation to ensure each feature followed a normal distribution. The edge attributes included the cosine similarity between the functional connectivity features that were calculated using the distance between the centers of the two ROIs. For each given node pair, their cosine similarity can be calculated as follows:

(7) $$S_{i,j}=\frac{x_{i}^{T}x_{j}}{\left\|x_{i}\|\|x_{j}\right\|}$$

The cross entropy loss function was utilized for the binary classification of sex. In Equation (8), N data points is where t is the truth value taking a value 0 or 1 and p is the softmax probability for the ith data point.

(8) $$L=-\frac{1}{N}\left[\sum_{j=1}^{N}\left[t_{j}\;log\left(p_{j}\right)+\left(1-t_{j}\right)log(1-pj)\right]\right.$$

### 2.5. Interpretability Using GNNExplainer

Furthermore, we applied GNNExplainer to interpret our model and identify significant functional connectivity subnetworks that played an important role in classifying sex. GNNExplainer is a model agnostic approach to providing explanations of classification results of a GNN-based model [30]. The interpretability method can provide consistent explanations of important node features that play a critical role in prediction. In order to identify the node features that are most important for prediction, GNNExplainer considers $X_{S}^{F}$ as a subset of features and learns a feature selector F, which acts as a feature mask, for nodes in the explanation of $G_{s}$ that are defined through the feature selector F∈{0,1} as follows:

(9) $$X_{S}^{F}=\left\{x_{j}^{F}|v_{j}\in G_{S}\right\},\;x_{j}^{F}=\left[x_{j,t1},\ldots ,x_{j,tk}\right]\;\text{for}\;F_{ti}=1$$

(10) $$\text{max}\;MI\left(Y,\left(G_{s},F\right)\right)=H(Y)-H\left(Y|G=G_{s},\;X=X_{S}^{F}\right)$$

In Equation (10) above, the explanation $\left(G_{s},X_{s}\right)$ is concurrently optimized to maximize the mutual information objective of $\left(G_{s},F\right)$, signifying the adjusted objective function that integrates structural and node feature data to elucidate the prediction $\overset{ˆ}{y}$ at node v [30]. The hyperparameters used for the method include prediction loss, feature size loss, feature element loss, population size loss, population element loss, weight decay, training epochs, and learning rate, with the values being 1, 200, 20, 0, 1000, 0, 150, and 5 × 10^−1^. After applying GNNExplainer and obtaining the results of the important subnetworks, we further found the common connections related to sex differences in each subnetwork.

The loss functions used in the GNNExplainer method include prediction loss, feature size loss, feature element loss, population size loss, and population element loss. Prediction loss measures the difference between the predicted probability of a positive outcome and the true label. For example, a common form of prediction loss is binary cross entropy loss, which penalizes incorrect predictions more heavily and rewards accurate predictions. Moreover, feature size loss is a loss term that penalizes changes in the size of the features for each node in the graph, and it is calculated as the mean squared error between the original feature size and the perturbed feature size after the GNNExplainer has made changes to the graph. Similarly, feature element loss is a loss term that penalizes changes to individual feature elements for each node in the graph. Population size loss is a loss term that penalizes changes in the overall size of the graph, which is calculated as the mean squared error between the original number of nodes and the perturbed number of nodes after the GNNExplainer has made changes to the graph. Population element loss is a loss term that penalizes changes to the edge weights or adjacency matrix of the graph, and it is calculated as the mean squared error between the original edge weights and the perturbed edge weights. The GNNExplainer algorithm iteratively minimizes these loss terms to find the most salient features and edges that contribute to the prediction outcome or the overall structure of the graph [30].

### 2.6. Experimental Setup and Sex Classification

In this study, the dataset was randomly divided into training, validation, and testing subsets, maintaining proportions of 80%, 10%, and 10%, respectively. The model was trained using the training set, and the hyperparameters were optimized using the validation set. The performance of the model was evaluated by calculating the accuracy, F1-score, and area under the curve (AUC) with standard deviation (std) values. To reduce sampling bias and evaluate the robustness of the models, we utilized the bootstrap analysis method, conducting 10 repeated experiments. Each experiment involved the splitting of the dataset, model training, and testing processes. We compared the results obtained from our proposed model with those of other approaches using the outcomes of the repeated experiments. A pairwise t-test was conducted, and p-values were reported to demonstrate any statistically significant improvement.

We applied MGIN to both working memory and emotion task fMRI and compared the performance with other models. After comparing the performance of our method, we performed sex classification during the five stages of adolescence to check whether our proposed framework can use functional connectivity as an effective brain fingerprint for prediction. Each experiment was repeated using 10-fold cross validation.

## 3. Results

### 3.1. Hyperparameter Selection

In order to optimize the performance of the MGIN model, the hyperparameters were tuned using the random search method on validation sets [40]. As shown in Table 3, the hyperparameters for the experiments included the learning rate, optimizer, epochs, weight decay, fMRI paradigms, and predictive task, with the values set as 1 × 10^−5^, Adam, 3000, 0.2, emoid/nback, and sex, respectively. The two-layer GINs in Equation (1) were used for the graph embedding process. The activation layer for the two-layer MGIN model is *ReLU*. The activation function was used to merge the graph embeddings of the two modalities.

In terms of hyperparameters, the validation of the single modal GIN was performed using emoid or nback fMRI datasets, utilizing the same hyperparameter selection that was applied in the GIN component of MGIN. For SVM and MLP, the hyperparameters were tuned using the random search method. For the MLP framework, we employed an identical network structure as the MGIN. Specifically, the hyperparameters for the MLP method consisted of 3000 training epochs, 1 × 10^−2^ as the L1 regularization parameter, 1 × 10^−3^ as the L2 regularization parameter, an Adam optimizer, and 1 × 10^−4^ as the learning rate. For the M-GCN framework, the hyperparameters included 5000 training epochs, 1 × 10^−4^ as the L2 regularization parameter, an Adam optimizer, and 1 × 10^−4^ as the learning rate. Additionally, for the MVGCN method, the hyperparameters we used were the Adam optimizer, a learning rate of 1 × 10^−4^, 3000 training epochs, and a L2 regularization parameter of 1 × 10^−4^.

The classification accuracy was evaluated using 10-fold cross validation and was run 10 times independently on the whole dataset. The p-values were calculated by the t-test for repeated experiments between the classification results of our MGIN model and other competing models. Our results showed that combining two paradigms of fMRI (emoid and nback) yielded higher performance than using a single paradigm, demonstrating the advantage of integrating multi-paradigm fMRIs.

### 3.2. Ablation Studies

For our ablation studies, we tested the effect of the number of modalities, number of layers, and node features on our proposed MGIN framework. As shown in Table 4, we compared the performance of MGIN with the use of different modalities. The accuracy (0.8167 ± 0.0749), F1-score (0.8192 ± 0.0683), and AUC (0.8269 ± 0.0754) were highest when utilizing both nback and emoid fMRI modalities. Additionally, we explored the effect of the number of hidden layers in our proposed MGIN method. Our experiments illustrated the optimum number of hidden layers is two with an accuracy of 0.8167 ± 0.074 and AUC of 0.8269 ± 0.075. Furthermore, our experimental results demonstrated the effect of various types of node features on the MGIN framework, where we found that the connection profile comprehensively captures the structural information of the brain network and retains extensive information on pairwise connections with an accuracy of 0.7012 ± 0.041, and it outperforms structural features like degree or positional features such as eigen decomposition.

### 3.3. Performance in Comparison to Other Methods

**Figure 4.**
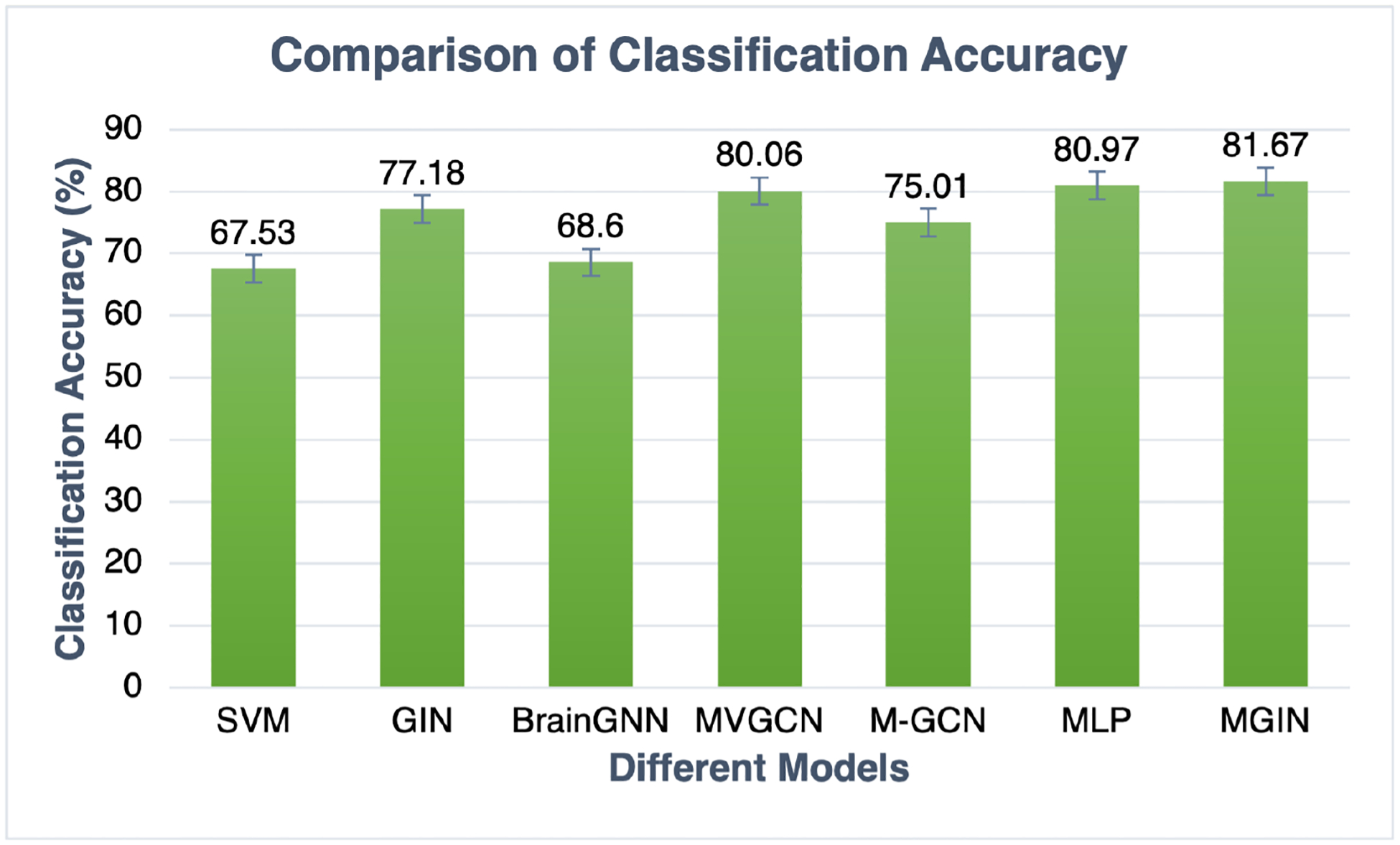
The comparison of classification accuracy by MGIN and six different algorithms.

Table 5 summarizes the performance of the seven algorithms tested on the PNC dataset. MGIN achieves the best classification performance in accuracy at 0.8167. MLP, a simple multi-layer perceptron model, obtains the second-best accuracy at 0.8097. The multi-view graph convolutional network, MVGCN, model achieves an accuracy of 0.8006, which is better than the results of other algorithms, e.g., an accuracy of 0.7718 for single paradigm GIN, an accuracy of 0.7501 for M-GCN, an accuracy of 0.6860 for BrainGNN, and an accuracy of 0.6753 for SVM. Compared to the other six algorithms, MGIN achieved the highest accuracy, as shown in Figure 4. Therefore, the experimental results of the PNC data reveal that the multi-modal graph isomorphism network indeed improves classification performance.

### 3.4. Model Explanation and Biomarker Identification

**Figure 5.**
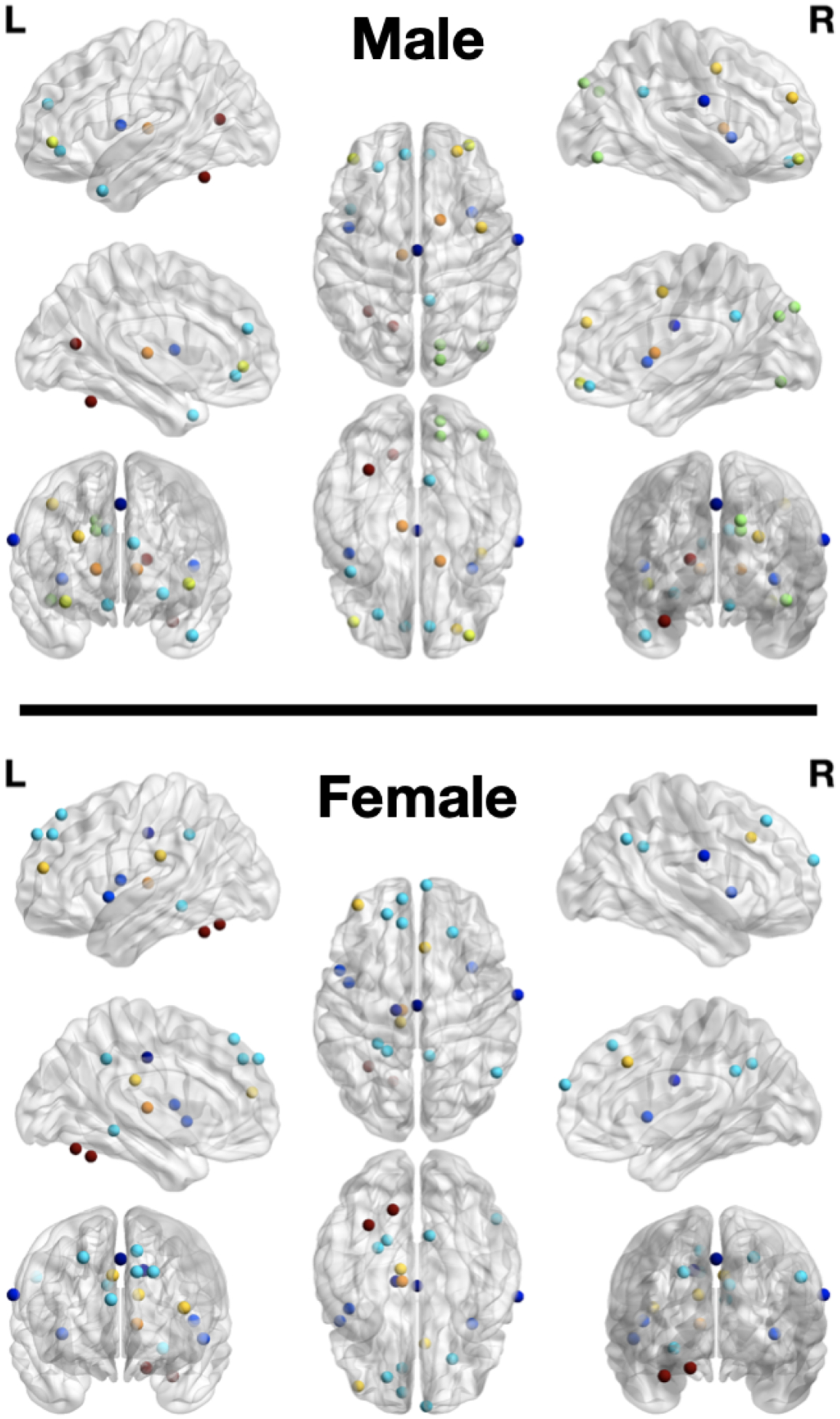
The top 5% of selected ROIs for female and male subjects based on the power264 template. The ROIs with the same color indicate that they are in the same functional network. The figure contains the different views of the brain (axial, coronal, and sagittal).

**Figure 6.**
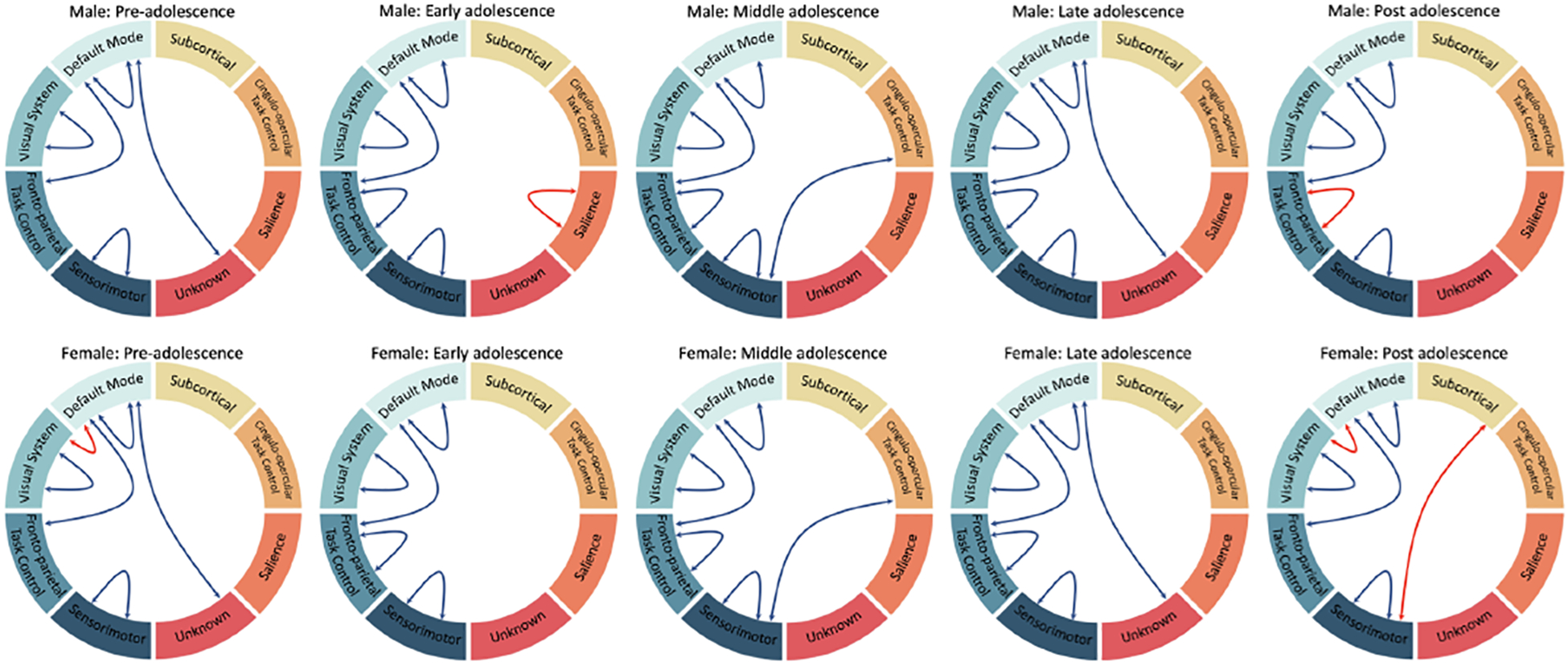
Important intra- and inter-network connections during the different stages of adolescence. In pre-adolescence, females have additional connections between the default mode and visual system. In early adolescence, males have intra-network connections in the salience network. In post-adolescence, females have additional connections between the default mode and visual system, subcortical and sensorimotor system, and fronto-parietal task control.

**Figure 7.**
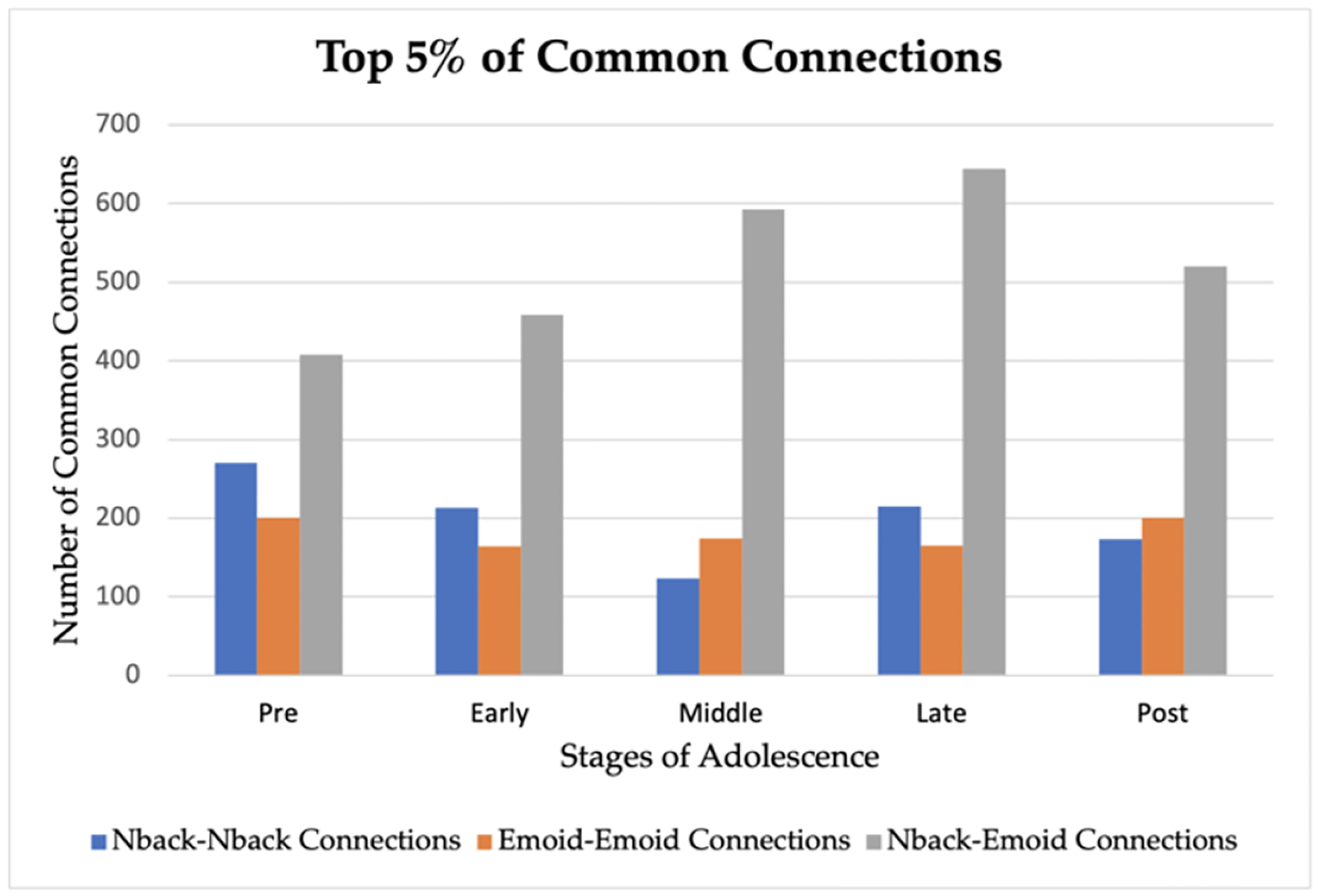
Top 5 percent number of common connections within and between MRI tasks.

We interpreted our model and identified the top 5% of common functional connections as important subnetworks using GNNExplainer using both working memory and emotion tasks for fMRI. In Table 6, we list the specific hyperparameters of GNNExplainer. The following abbreviations of the functional networks were used: sensorimotor (SM Hand and SM Mouth), visual (VIS), default mode (DMN), fronto-parietal task control (FRNT), cerebellum (CB), and salience (SAL). The number of common functional connections varied during each of the five stages of adolescence. In Table 7, we identified the top 5% of most important connections for sex classification in adolescence. Additionally, in Figure 5, the specific top 5% of important selected regions of interest can be visualized in the axial, coronal, and sagittal views. Furthermore, in Figure 6, the critical subnetworks associated with sex variation were identified. In Figure 7, we identified the number of top 5% of common connections within and between MRI tasks during the various stages.

## 4. Discussion

In our work, we proposed an interpretable MGIN framework in order to classify the sex of a subject during the various stages of adolescence using both the emoid and nback fMRI tasks, which can help us understand the sex differences in brain development. We ultimately validated our model using the PNC dataset, and our results demonstrated that MGIN had improved performance compared to state-of-the-art algorithms in deep learning. Based on our ablation experiment, we found that the accuracy decreases when the number of layers is increased. Several plausible explanations exist for the observed decline in accuracy upon increasing the number of hidden layers to three. One potential rationale revolves around overfitting, wherein augmenting the neural network’s layers may escalate its capacity, particularly when confronted with a small and noisy dataset. The consequential surge in hidden layers beyond an optimal threshold can precipitate a decline in test set accuracy, indicative of the network’s propensity to overfit to the training set. Consequently, while the network may adeptly learn the intricacies of the training data, its ability to generalize to novel and unseen data diminishes. Moreover, each additional layer exacerbates the computational burden of training and inference, potentially prolonging training times and heightening computational resource requisites, particularly if the network architecture surpasses the requisite complexity for the designated task. Hence, to optimize the performance of the MGIN model in our experiments, we undertook hyperparameter optimization through the random search method on validation sets [36,37,40,41].

Next, we applied GNNExplainer to interpret our model as well as to identify the significant ROIs and subnetworks for functional connectivity that played a critical role in classifying sex. Based on the important ROIs we identified, we were able to identify the critical subnetworks related to sex variability. Sex-related differences in the functional subnetworks have been identified by previous studies utilizing fMRI [7,42,43]. Previous approaches have utilized multivariate pattern analysis using support vector machines [7,42] and a parameter-free centralized multi-task learning method [43] to understand the resting state functional connectivity (RFSC) patterns that are predictive for brain development to aid in understanding sex differences.

### 4.1. Differences in Intra-Network Connections

- Default Mode Network (DMN)
  According to previous studies, DMN is a signature intrinsic network that is frequently linked to sex differences [44]. For example, in the DMN, the basal configuration exhibits distinct sex-specific dynamics, and it diverges between the sexes during early adulthood. There is a globally age-modulated reconfiguration in men but not women, where the reconfiguration correlates with measures of personality traits. In women, the basal reconfiguration likely exhibits a strong dependence on the menstrual cycle [45].
- Sensorimotor (SM Hand Mouth)
  Based on previous studies, females spend more time than males in two transient network states that spatially overlap with the sensory motor network and dorsal attention network [46].
- Cingulo-Opercular Task Control (CNG)
  Additionally, men and women have different functional connectivity patterns. For the regulation of emotion, a better suppression of negative emotion in women was associated with stronger functional connectivity in CNG [46–48].
- Subcortical (SUB)
  Studies have shown substantial functional connectivity differences between the sexes in several subcortical regions including the amygdala, caudate, thalamus, and cortical regions such as the inferior frontal gyrus [47].
- Visual System (VIS)
  Previous research has shown various differences in the visual system across the sexes, as females tend to prioritize the utilization of low spatial frequencies, which convey information regarding the overall structure of objects, while males exhibit a segregative approach that emphasizes individual objects and intricate details [49].
- Fronto-Parietal Task Control (FRNT)
  In the FRNT, women have shown higher connectivity than men in the left middle frontal gyrus (MFG) for the anterior network, and another cluster is found in the right MFG right dorsal network. Previous resting state fMRI studies have shown that women have higher connectivity in prefrontal regions for cognitive networks, which includes the IFG, MFG, and medial prefrontal regions [50].
- Salience (SAL)
  The salience network, a network associated with helping direct attention to the most relevant stimuli in one’s environment, has differences between male and female subjects. For example, in male patients with autism, they had increased connectivity between the salience and primary sensory networks [51].

### 4.2. Differences in Inter-Network Connections

- Visual System (VIS) ↔ Default Mode Network (DMN)
  In our study, we found that females have additional inter-network connections between the visual system and default mode network during the pre-adolescent stage. Specifically, there are 94 connections between these subnetworks in pre-adolescence. Based on previous studies, females have shown greater hyperconnectivity in the DMN compared to males [44,45,49]. Our work provides further insight into where the subnetwork connections exist during different stages of adolescence.
- Subcortical (SUB) ↔ Sensorimotor (SM Hand Mouth)
  During post-adolescence, females have additional connections between the subcortical and sensorimotor networks. In particular, there are 65 connections between these subnetworks that are not seen in males. Previous research has shown the substantial differences in sex for the SUB network and SM Hand Mouth [46–48], but our work provides further knowledge about the types of connections that exist.

### 4.3. Limitations

Our approach possesses several limitations that warrant consideration. First, although our model adeptly captures the intricate non-linear relationships between variables and labels, it neglects to incorporate dimensionality reduction techniques to address the diverse nature of high-dimensional datasets within the population. To remedy this deficiency, we intend to implement various dimensionality reduction methods in future iterations of our research. Second, within our graphical learning framework, the features are predicated upon the Pearson correlation coefficient, while the edge weights are derived from the cosine similarity between functional connectivity features among nodes. While these choices render our graphical network amenable to learning, we recognize the potential for improvement through the exploration of alternative feature learning and sparsity techniques, aiming to diversify edge weights and mitigate redundancy in high-order relationships. Third, in the context of our neurodevelopmental application, our study concentrates on delineating significant subnetworks and crucial ROIs contributing to sex-based distinctions. In forthcoming investigations, we plan to extend our inquiry to encompass variances in connectomes across age cohorts, developmental stages, and cognitive groupings, thereby augmenting our understanding of neurodevelopmental processes and associated biological pathways.

Furthermore, despite the generalizability of our framework to analyze diverse neuroimaging data and disease contexts, it is imperative to acknowledge the potential influence of factors such as demographic heterogeneity, variability in disease phenotypes, and variations in data acquisition protocols on generalization performance. Our model may encounter challenges in extending its predictive capacity to populations characterized by dissimilar demographic compositions, disease prevalence rates, or cultural influences not adequately represented in the training dataset. Additionally, the inherent heterogeneity of multi-modal datasets, encompassing a breadth of information types including imaging, clinical, genetic, and demographic data, poses a notable challenge in ensuring the equitable contribution and relevance of each modality across diverse populations. To address this, we propose the application of graph fusion techniques, facilitating the amalgamation of information derived from multiple graphs representing distinct modalities. This entails the exploration of methods such as learning separate embeddings for each modality and subsequently integrating them via concatenation, summation, or attention mechanisms. In the future, we aim to develop a unified representation capable of encapsulating information from all modalities concurrently [52–55].

Zhang et al. conducted a comprehensive examination of recent advancements in theranostics tailored for MRI-guided therapy, with a particular focus on the integration of therapeutic and diagnostic facets within MRI-guided procedures. While our current research is centered on the utilization of fMRI data exclusively, the potential synergy between theranostics for MRI-guided therapy and fMRI offers several compelling prospects for both research and clinical practice. One such prospect involves harnessing fMRI for the real-time monitoring of therapeutic interventions. By amalgamating MRI-guided therapy with fMRI, investigators and clinicians can evaluate alterations in brain activity or physiological responses during treatment, thereby facilitating personalized treatment optimization. Moreover, fMRI can serve as a valuable tool for probing the underlying mechanisms of therapeutic interventions through the longitudinal monitoring of changes in brain activity patterns or connectivity subsequent to treatment. This integrated approach offers the potential to deepen our understanding of brain function, disease pathology, and treatment responses, ultimately paving the way for more efficacious personalized interventions tailored to neurological and psychiatric disorders [56].

## 5. Conclusions

In this work, we proposed an interpretable MGIN model for the joint analysis of multi-paradigm fMRI data. Our MGIN model can achieve better performance in understanding sex differences and identifying important underlying functional networks when compared to other baseline methods. We applied the MGIN model to a brain imaging cohort to classify individuals by sex during different stages of adolescence, and our experimental results showed that our multi-modal model can deliver improved accuracy over the use of single modality when classifying sex groups. Our model identified the most important sex-related functional networks, which include DMN, VIS, CNG, FRNT, SAL, SUB, and SM Hand Mouth. By investigating the common connections among the multi-paradigm data, our study reveals important subnetworks related to sex differences across adolescence [57–66]. Therefore, the method offers a way of understanding sex differences in neurodevelopment and providing guidance on early intervention [2,4,24]. Finally, our framework is generic and generalizable to the analysis of other neuroimaging data and diseases.

## Figures and Tables

**Table 1.** Frequently used motation.

Notation	Description
G=(V,E)	Graph with V vertices and E edges
${\text{X}}_{v}$	Node feature vector for v∈V
${\text{y}}_{v}$	Associated label to node v∈V
${\text{h}}_{v}$	Representation vector of v∈V
$\left\{{\text{G}}_{1},\ldots ,{\text{G}}_{N}\right\}\subset \text{G}$	Set of graphs
$\left\{{\text{y}}_{1},\ldots ,{\text{y}}_{N}\right\}\subset \text{Y}$	Set of labels
${\text{h}}_{G}$	Representation vector
${\text{y}}_{G}=\text{g}\left({\text{h}}_{G}\right)$	Predicted label of an entire graph
k	kth layer of a GNN (kth iteration)
${\text{h}}_{G}=\text{READOUT}$	Summation or group-level pooling function

**Table 2.** Distribution of subjects in adolescence.

Adolescent Stage	Age Range	Total Subjects	Sex Distribution
Pre	8–12	129	55 male/74 female
Early	12–14	113	58 male/55 female
Middle	14–16	109	54 male/55 female
Late	16–18	130	59 male/71 female
Post	18–22	141	53 male/88 female

**Table 3.** Hyperparameters for experiments.

Learning Rate	1 × 10^−5^
Optimizer	Adam
Epochs	3000
Layer 1 Size	69,696
Layer 2 Size	100
Weight Decay	0.2
fMRI Paradigms	Emoid, Nback
Predictive Task	Sex

**Table 4.** Ablation studies.

	Method	Accuracy (Mean ± std)	F1 (Mean ± std)	AUC (Mean ± std)
*Modalities*	1 Modality Nback	0.7814 ± 0.0722	0.7801 ± 0.0568	0.7995 ± 0.0453
	1 Modality Emoid	0.7621 ± 0.0501	0.7721 ± 0.0452	0.7861 ± 0.0834
	2 Modality Nback+Emoid	0.8167 ± 0.0749	0.8192 ± 0.0683	0.8269 ± 0.0754
*Layers*	1 Hidden Layer	0.8152 ± 0.0537	0.8110 ± 0.0764	0.8237 ± 0.0472
	2 Hidden Layers	0.8167 ± 0.0749	0.8192 ± 0.0683	0.8269 ± 0.0754
	3 Hidden Layers	0.8092 ± 0.0438	0.8014 ± 0.0467	0.8137 ± 0.0564
*Node Features*	Eigen	0.5140 ± 0.0392	0.4934 ± 0.0562	0.5018 ± 0.0754
	Degree	0.6389 ± 0.0227	0.6023 ± 0.0385	0.7025 ± 0.0437
	Connection profile	0.7012 ± 0.0416	0.6621 ± 0.0476	0.7670 ± 0.0549

All of the p-values were p≤0.05 for the test metrics and were computed using a t-test. The standard deviation is represented by “std”.

**Table 5.** The comparison of the performance of different models on the PNC dataset.

Model	Modalities	Accuracy (Mean ± std)	p-Value	F1 (Mean ± std)	p-Value	AUC (Mean ± std)	p-Value
SVM	Emoid fMRI	0.6801 ± 0.0824	3.58 × 10^−8^	0.6912 ± 0.0542	2.58 × 10^−2^	0.7091 ± 0.0643	1.92 × 10^−8^
SVM	Nback fMRI	0.6704 ± 0.0818	2.98 × 10^−9^	0.6781 ± 0.0823	3.01 × 10^−5^	0.6847 ± 0.0567	2.32 × 10^−9^
GIN	Emoid fMRI	0.7621 ± 0.0501	0.0219	0.7721 ± 0.0452	0.0154	0.7861 ± 0.0834	0.0322
GIN	Nback fMRI	0.7814 ± 0.0722	0.1380	0.7801 ± 0.0568	0.1125	0.7995 ± 0.0453	0.0929
MLP	Emoid fMRI & Nback fMRI	0.8097 ± 0.0944	0.1131	0.8085 ± 0.0756	0.1026	0.8109 ± 0.0985	0.0932
BrainGNN	Emoid fMRI & Nback fMRI	0.6860 ± 0.0829	0.1121	0.6899 ± 0.0756	0.1729	0.7022 ± 0.0563	0.2382
MVGCN	Emoid fMRI & Nback fMRI	0.8006 ± 0.0504	0.4730	0.8093 ± 0.0583	0.4372	0.8135 ± 0.0731	0.4572
M-GCN	Emoid fMRI & Nback fMRI	0.7501 ± 0.0504	0.1131	0.7525 ± 0.0431	0.1092	0.7721 ± 0.0621	0.1356
MGIN *	Emoid fMRI & Nback fMRI	0.8167 ± 0.0749	-	0.8192 ± 0.0683	-	0.8269 ± 0.0754	-

* The p-values were computed using a t-test to compare the regression performance of our MGIN model, in repeated experiments, with that of other competing models. The standard deviation is represented by “std”.

**Table 6.** Hyperparameters for GNNExplainer.

Prediction Loss	1
Feature Size Loss	200
Feature Element Loss	20
Population Size Loss	0
Population Element Loss	1000
Weight Decay	0
Training Epochs	150
Learning Rate	5 × 10^−1^

**Table 7.** The top 5% of most important connections for sex classification in adolescence.

Modality	Sex	ROI	ROI Region	MNI Space	FN
*Nback*	Female	2	Left Cerebrum | Limbic Lobe | Cingulate Gyrus	−14 −18 40	SM Hand
		35	Right Cerebrum | Frontal Lobe | Precentral Gyrus	66 −8 25	SM Mouth
		44	Left Cerebrum | Frontal Lobe | Precentral Gyrus	−45 0 9	CNG
		79	Right Cerebrum | Limbic Lobe | Cingulate Gyrus	8 −48 31	DMN
		84	Right Cerebrum | Frontal Lobe | Middle Frontal Gyrus	23 33 48	DMN
		85	Left Cerebrum | Frontal Lobe | Superior Frontal Gyrus	−10 39 52	DMN
		90	Left Cerebrum | Frontal Lobe | Superior Frontal Gyrus	−10 55 39	DMN
		91 *	Left Cerebrum | Frontal Lobe | Superior Frontal Gyrus	−20 −45 39	DMN
		111	Right Cerebrum | Limbic Lobe | Parahippocampa Gyrus	−26 −40 −8	DMN
		199	Left Cerebrum | Frontal Lobe | Superior Frontal Gyrus	−39 51 17	SAL
	Male	35	Right Cerebrum | Frontal Lobe | Precentral Gyrus	66 −8 25	SM Mouth
		44	Left Cerebrum | Frontal Lobe | Precentral Gyrus	−45 0 9	CNG
		65	Right Cerebrum | Frontal Lobe | Medial Frontal Gyrus	8 48 −15	DMN
		70	Left Cerebrum | Temporal Lobe | Superior Temporal Gyrus	−44 12 −34	DMN
		79	Right Cerebrum | Limbic Lobe | Cingulate Gyrus	8 −48 31	DMN
		102	Left Cerebrum | Frontal Lobe | Medial Frontal Gyrus	−8 48 23	DMN
		111	Left Cerebrum | Limbic Lobe | Parahippocampa Gyrus	−26 40 −8	DMN
		142	Left Cerebrum | Occipital Lobe | Lingual Gyrus	15 −77 31	VIS
		164	Right Cerebrum | Frontal Lobe | Middle Frontal Gyrus	34 54 −13	FRNT
		177	Left Cerebrum | Frontal Lobe | Middle Frontal Lobule	−42 45 −2	FRNT
*Emoid*	Female	3	Left Cerebrum | Frontal Lobe | Paracentral Lobule	0 −15 47	SM Hand
		47	Left Cerebrum | Temporal Lobe | Superior Temporal Gyrus	−51 8 −2	CNG
		49	Right Cerebrum | Sub-lobar | Insula	36 10 1	CNG
		83	Right Cerebrum | Parietal Lobe | Angular Gyrus	52 −59 36	DMN
		91 *	Left Cerebrum | Frontal Lobe | Superior Frontal Gyrus	−20 45 39	DMN
		93	Left Cerebrum | Frontal Lobe | Medial Frontal Gyrus	6 64 22	DMN
		191	Left Cerebrum | Limbic Lobe | Anterior Cingulate	−11 26 25	SAL
		195	Right Cerebrum | Frontal Lobe | Cingulate Gyrus	5 23 37	SAL
		202	Left Cerebrum | Sub-lobar | Thalamus	−10 −18 7	SUB
		233	Left Cerebellum | Cerebellum Posterior Lobe | Declive	−16 −65 −20	CB
		234	Left Cerebellum | Cerebellum Posterior Lobe | Culmen	−32 −55 −25	CB
	Male	3	Left Cerebrum | Frontal Lobe | Paracentral Lobule	0 −15 47	SM Hand
		49	Right Cerebrum | Sub-lobar | Insula	36 10 1	CNG
		136	Right Cerebrum | Occipital Lobe | Inferior Occipital Gyrus	43 −78 −12	VIS
		139	Right Cerebrum | Parietal Lobe | Precuneus	15 −87 37	VIS
		184	Right Cerebrum | Frontal Lobe | Middle Frontal Gyrus	42 0 47	SAL
		198	Right Cerebrum | Frontal Lobe | Superior Frontal Gyrus	26 50 27	SAL
		202	Left Cerebrum | Sub-lobar | Thalamus	−10 −18 7	SUB
		211	Right Cerebrum | Sub-lobar | Extra-Nuclear	15 5 7	SUB
		233	Left Cerebellum | Cerebellum Posterior Lobe | Declive	−16 −65 −20	CB
		234	Left Cerebellum | Cerebellum Anterior Lobe | Culmen	−32 −55 −25	CB

CNG: cingulo-opercular task control; SM Hand Mouth: sensorimotor; DMN: default mode network; VIS: visual; FRNT: fronto-parietal task control; SAL: salience; SUB: subcortical; CB: cerebellum; UNK: unknown

* Represents the ROIs common to both nback and emoid fMRI tasks

## Data Availability

Data available in a publicly accessible repository.
